# Platinum-Based Versus Non-Platinum-Based Chemotherapy as First Line Treatment of Inoperable, Advanced Gastric Adenocarcinoma: A Meta-Analysis

**DOI:** 10.1371/journal.pone.0068974

**Published:** 2013-07-11

**Authors:** Wei-Wei Chen, Feng Wang, Rui-Hua Xu

**Affiliations:** Department of Medical Oncology and State Key Laboratory of Oncology in South China, Sun Yat-sen University Cancer Center, Guangzhou, Guangdong, China; Vanderbilt University Medical Center, United States of America

## Abstract

**Background:**

Although the platinum regimen is adopted widely nowadays in spite of the excessive side effects, there is still no international standard for palliative chemotherapy of advanced gastric cancer. This meta-analysis assessed the efficacy and tolerability of platinum versus non–platinum chemotherapy as first-line palliative treatment in patients with inoperable, advanced gastric cancer.

**Methods:**

Randomized phase II and III clinical trials on first-line palliative chemotherapy in inoperable, advanced gastric cancer were identified by electronic searches of PubMed, Embase, and the Cochrane Controlled Trial Register, and hand searches of relevant abstract books and reference lists. Response rates, overall survival, and toxicity were analyzed. Depending on whether new-generation agents (S-1, taxanes and irinotecan) were utilized, the non–platinum regimens were divided into two subgroup.

**Results:**

Compared to non-platinum regimens containing new-generation agents, the use of platinum-based regimens was associated with better response (risk ratio (RR) = 1.94, 95%CI[1.48, 2.55], p<0.001), an increase of overall survival (hazard ratio (HR) = 0.85, 95%CI[0.78, 0.92], p<0.001), a higher risk of hematological and non-hematological toxicity. No statistically significant increase in response (RR = 1.03, 95%CI [0.85, 1.24], p = 0.76) or overall survival (HR = 1.07, 95%CI [0.88, 1.30], p = 0.49) was found when platinum therapies were compared to new-generation agent based combination regimens. The toxicity of platinum-based regimens was significantly higher for hematologic toxicity, nausea and vomiting, and neurotoxicity, but not for diarrhea and toxic death rate.

**Conclusion:**

New-generation agent based combination regimens achieved similar response rate and overall survival as platinum-based therapy that had generally higher side effects. S-1, taxanes and irinotecan seemed to be valid options for patients with inoperable, advanced gastric cancer as first-line chemotherapy.

## Introduction

Although the mortality rates for gastric cancer has been declining after the late 1940s [1], gastric cancer still remains the second leading cause of cancer related death all over the world with current overall 5-year survival rates less than 20% [2]. Because of silent symptoms in the early stages, many patients are diagnosed when their disease is already inoperable or advanced, which makes them lose the opportunity of radical surgery. For these patients, the objectives of treatment are to relieve symptoms, prevent tumor progression and prolong survival. A meta-analysis published in 2006 has confirmed the value of palliative chemotherapy by comparison with best supportive care [3]. The early regimens such as fluorouracil-adriamycin-methotrexate (FAMTX), etoposide-leucovorin-fluorouracil (ELF), fluorouracil-adriamycin-mitomycin (FAM), were compared to cisplatin-based regimens in several clinical controlled trials but the conclusions were reported controversially both in response rate and overall survival [4]–[19]. At the end of the 1990s, platinum-based chemotherapy regimens such as cisplatin-5-fluorouracil (CF), epirubicin-cisplatin- 5-fluorouracil (ECF) were widely used in western European countries but not in United States [20], [21], though platinum seemed to decrease the life quality because of substantially worse toxicity. Although S-1, irinotecan and taxanes were developed as new-generation agents and many phase II and phase III clinical trials have been conducted to compare them to traditional platinum-based regimens in the first-line therapy [6], [7], [22]–[31], the results are conflicting and no definitive conclusion about the superiority of either regimen is drawn. In addition, so far there are no international standard regimens for palliative chemotherapy of advanced gastric cancer. Therefore, it was deemed important to perform this meta-analysis of randomized phase II and III treatment trials for comparing the efficiency, tolerance and prognosis between platinum-based and non-platinum-based therapies in patients with inoperable advanced gastric cancer.

## Methods

Data from PubMed (MEDLINE, Old Medline and others), Embase, and the Cochrane Controlled Trial Register, from 1988 to August 2012, was sought electronically, including both full texts and abstracts. The strategy filter used MESH terms of “stomach neoplasms,” “antineoplastic agents,”“drug therapy,”“chemotherapy,” and “palliative care” (search strings in supplementary materials). No restriction on language of publication was considered. Initial data search was performed in August 2012, updating was in November 2012. To ensure that all relevant trials were included, we also performed manual searches in reference lists and conference proceedings of American Society of Clinical Oncology (ASCO), the European Conference of Clinical Oncology (ECCO) and the European Society for Medical Oncology (ESMO), from 1998 to 2006. Results were double checked by two independent reviewers. Authors were contacted to obtain any missing information and/or updates by e-mail.

### Eligibility Criteria

To be included in this meta-analysis, trials had to fulfill the following criteria: (1) being a randomized controlled phase II or phase III trial, which included patients suffering from histological confirmed, inoperable, advanced, or recurrent adenocarcinoma of the stomach or gastroesophageal junction. (2) with patients receiving regimens which compared platinum-based regimens with non-platinum-based regimens given as first-line palliative chemotherapy. The exclusion criteria were as follows: (1) letters, reviews or case reports were excluded; (2) non-prospective trials were excluded.

### Data Extraction

Two independent reviewers collected data from the identified trials: date of publication, form of publication, regimens used, dose used, number of patients assembled for response, hazard ratio (HR) with its 95% confidence interval (CI) for overall survival, number of patients assembled for toxicity, percent of patients with WHO or NCICTCAE grade 3 and 4 toxicities which included anemia, neutropenia, thrombocytopenia, febrile neutropenia, nausea and vomiting, diarrhea, neurotoxicity, nephrotoxicity and toxic death.

### Validity Assessment

The risk of confounding and the design quality of selected studies were qualitatively evaluated by two reviewers. Methodological quality of all eligible randomized controlled trials was assessed by the Cochrane Collaboration’s tool for assessing risk of bias. This quality result was used as the basis for sensitivity analysis.

### Statistical Analysis

We executed and reported our findings according to Preferred Reporting Items for Systematic Reviews and Meta-Analyses (PRISMA) statements [32], [33]. This analysis was calculated by Review Manager 5.1 developed by Cochrane Collaboration. The pooled estimates of response to treatment and toxicity used relative risk and its 95% confidence interval. Overall survival was defined as the time from randomization to death or to the last follow-up which was used as a date of censoring. The overall survival analysis was calculated with HR and 95%CI for HR. The heterogeneity (defined by different chemotherapy regimens) between studies was tested by X^2^ test, and measured with I^2^ statics (I^2^ = 66%). In order to reduce heterogeneity, trials were stratified into 2 subgroups depending on whether new-generation agents (S-1, taxanes or irinotecan) were contained in non-platinum arm. Because of heterogeneity existing in our analysis, the DerSimonian-Laird random effects model was chosen. Funnel plot was used to assess publication bias.

Seven trials randomized patients in three arms and one trials randomized patients in four arms [6], [7], [10], [12], [15], [16], [19], [30]. In three-arm designed trials, the number of patients, which used twice in analysis, was divided by two. Similarly, in the four-arm trial, the number of patients in non-platinum arm entered the analysis three times and was divided by three. This can deal with the increased influence on overall result. Overall survival was the primary outcome measure. An estimate of overall survival which is a time-to-event outcome was considered to use HR. For those papers, which did not publish HR directly, HR and 95%CI of HR for overall survival were extracted from the Kaplan-Meier survival curve by computer [34].

WHO criteria for response was widely used in numerous of trials before 2005 while RECIST criteria were extensively adopted in clinical trials after 2007.Actually, RECIST criteria are comparable to the old response criteria (WHO) in evaluating response in solid tumors [35].So we conducted pooled estimates of response ignoring the different criteria for evaluating response. Trials can be used in estimates of toxicity when they reported side effect in form of percentage of treated patients who experienced such toxicity, otherwise they were excluded from analysis of toxic effect.

## Results

### Literature Search

**Figure 1:**
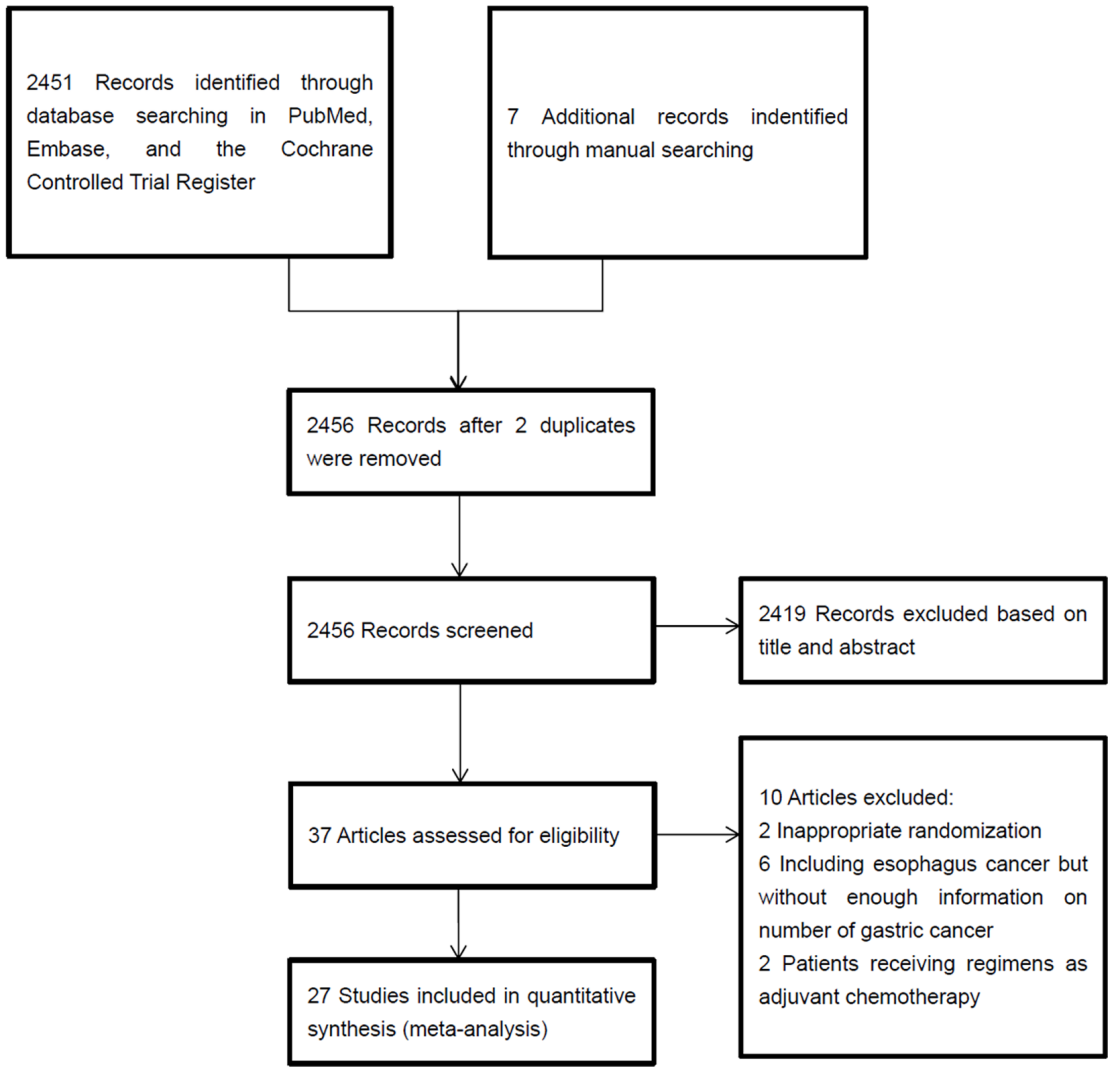
Selection of Articles for Meta-Analysis.

Our flow chart of the paper screening is shown in Figure 1. 39 randomized trials were retained for full review. 2 trials were excluded from the data collection because of inappropriate randomization. 6 trials, which included both stomach and esophagus cancer, were excluded, because of unsufficient available information about number of stomach cancer [36]–[38]. Additionally, results of 2 articles were too preliminary to be included. Other 27 evaluated trials were fully reviewed and data were collected from them. Among these trials 25 were fully published, and the other 2 were published in abstract form [29], [39]. Assessing risk of bias for all eligible randomized controlled trials can be seen in Figure S1.The characteristics of the trials and regimens are shown (Table S1). A total of 3680 patients were included in the 27 trials: 1587 patients were randomized in platinum-based regimens, while 2093 patients in non-platinum-based regimens. The response data was available in 26 studies. HR and 95% confidence interval of survival were available in 18 studies. Indeed, most of studies had survival curves. For those studiess which did not report the HR and 95% CI, we extracted data from survival curve and calculated HR and 95% CI indirectly by computer [34]. The grade 3 or 4 toxic effect was reported heterogeneously (16 for anemia, 14 for neutropenia, 15 for thrombocytopenia, 7 for nephrotoxicity, 19 for nausea and vomiting, 17 for diarrhea, 16 for neurotoxicity, 5 for toxic death).

### Response

**Figure 2:**
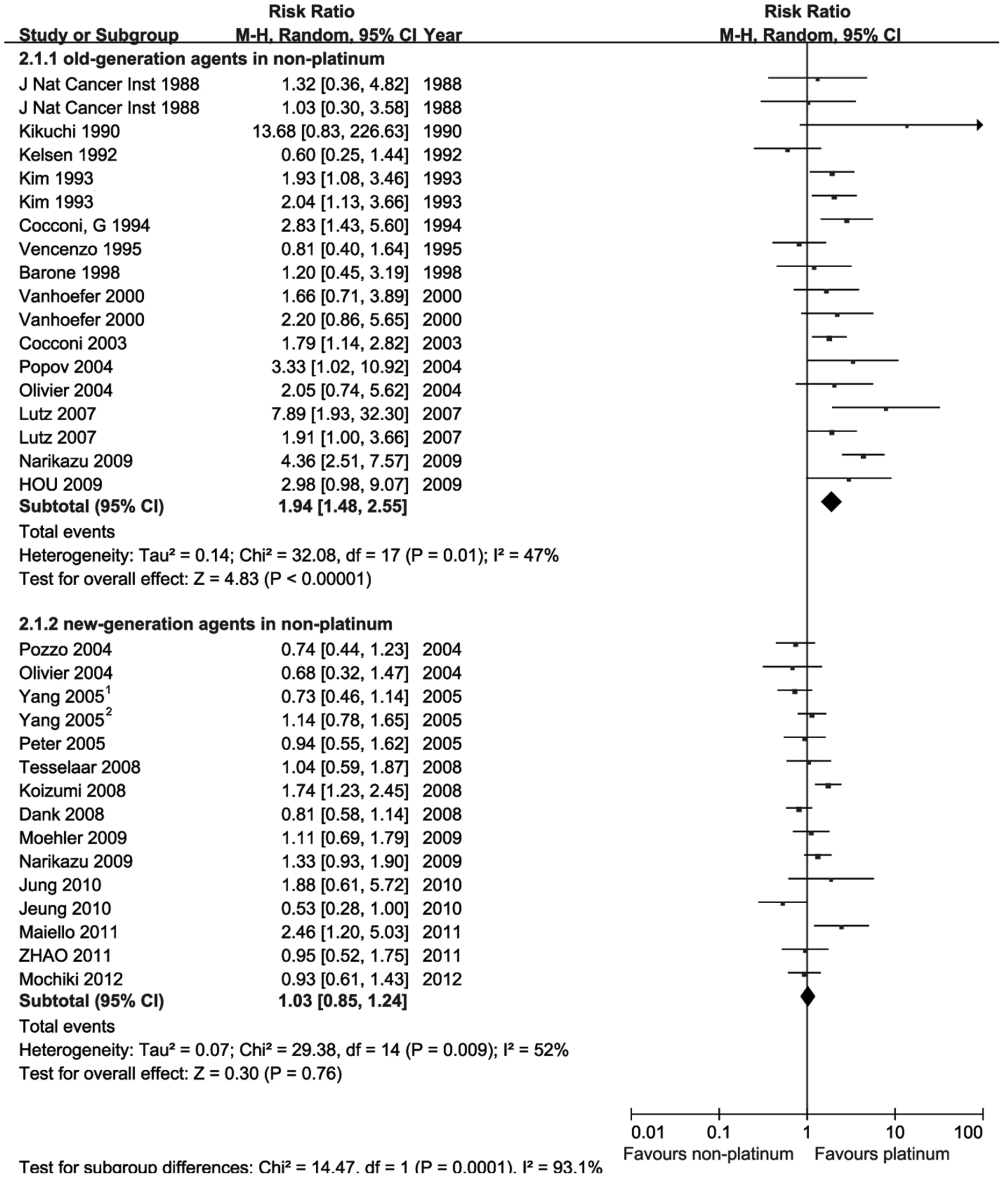
Comparison of response rate between platinum-based therapy and nonplatinum-based therapy. Subgroups were stratified according to whether the non-platinum contained new-generation agents such as S-1, taxanes or irinotecan.

Two subgroups were stratified from the non-platinum group depending on whether new-generation agents such as S-1, taxol or irinotecan are contained. Information available for response was stated in 26 trials. Each subgroup included 15 pair-wise comparisons. Two forest plots and pooled estimates of response were shown in Figure 2. 3428 patients were assembled for response analysis. When the response of platinum-based therapies was compared with old-generation therapies, the response seems to be higher in patients receiving platinum-based regimens (Risk Ratio (RR) = 1.94, 95% CI[1.48, 2.55], p<0.001).

Contrary to the results above, the platinum-based regimens did not seem to be associated with increased response compared to non-platinum regimens containing new-generation agents (RR = 1.03, 95%CI [0.85, 1.24], p = 0.76).No obvious benefit can be seen in favor of patients receiving platinum regimens. Test for subgroup differences was also proceeded (I^2^ = 93.1%, p = 0.001), showing the significant difference in heterogeneity between two subgroups.

### Survival

**Figure 3:**
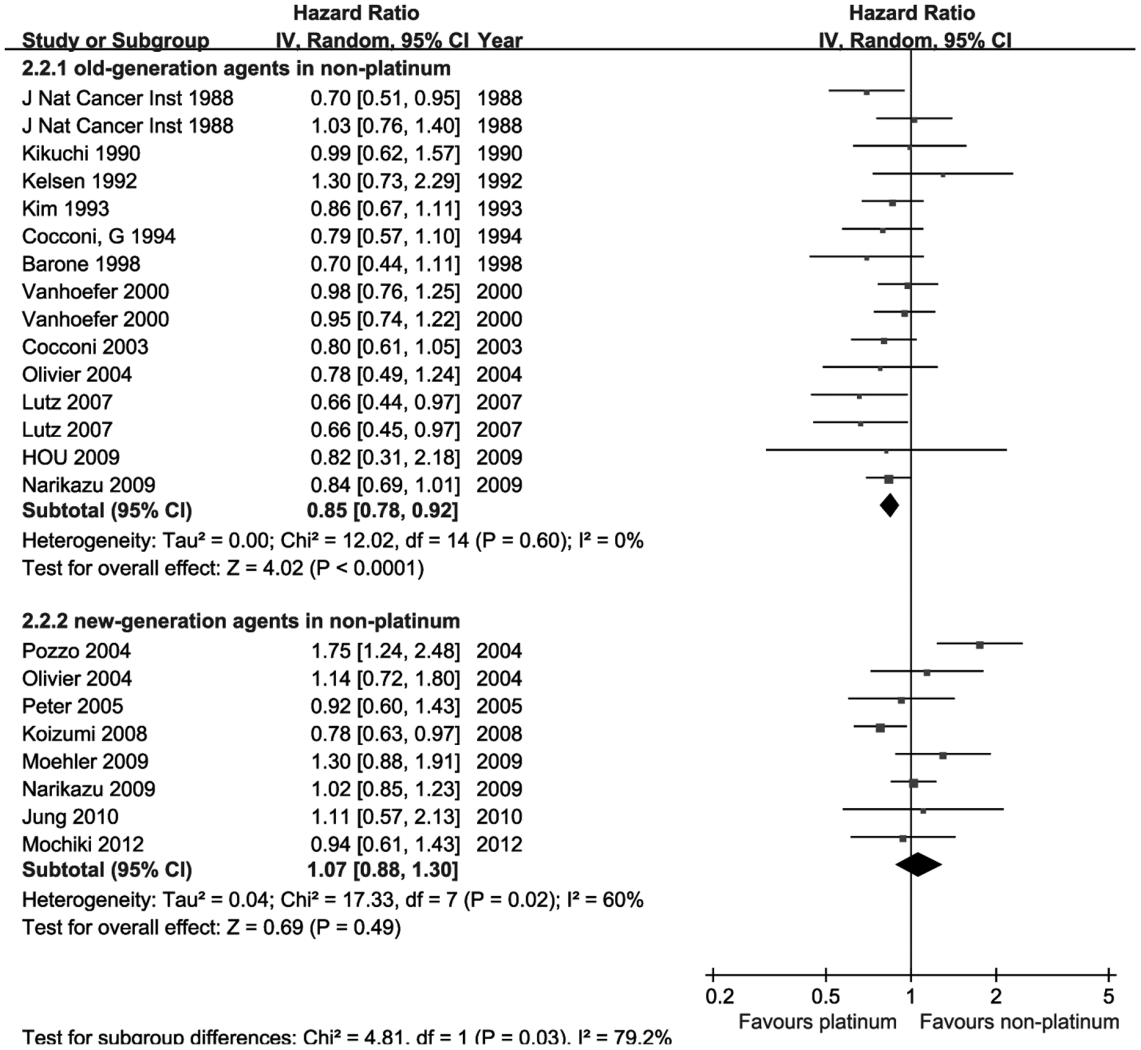
Comparison of overall survival between platinum-based therapy and nonplatinum-based therapy. Subgroups were stratified according to whether the non-platinum contained new-generation agents such as S-1, taxanes or irinotecan.

In this meta-analysis, 18 studies were available for survival analysis and subgroup analysis was also conducted. Two forest plots presented the results of pooled estimates in Figure 3. When comparison of platinum containing regimens versus non-platinum therapies without new-generation agents were conducted, the pooled HR was 0.85 (95%CI [0.78, 0.92], Z = 4.02, p<0.001), which illustrate that the platinum-based regimens had a better overall survival.

To further investigate the difference between platinum-based therapies and non-platinum-based therapies containing new-generation agents, 8 trials were enrolled in pooled estimates of overall survival. The pooled HR was 1.07 (95%CI [0.88, 1.30], Z = 0.69, p = 0.49), which illustrated that the platinum-based regimens were not significantly associated with increased overall survival compared to new-generation agents containing regimens. This subgroup finding was similar to the result of response analysis above.

### Toxicity

Side effects were analyzed as WHO (or NCICTCNE) grade 3 and 4 toxicity combined, using data reported in a way of toxicities in a per-patient fashion. When the comparison was restricted to the trials using the old generational combination in non-platinum arm, the platinum was found to be associated with a higher toxic effect for anemia (RR = 1.93, 95%CI, 1.53–2.44, p<0.001), neutropenia (RR = 30.67, 95%CI, 16.92–55.57, p<0.001), febrile neutropenia (RR = 9.05, 95%CI, 3.64–22.50, p<0.001), nausea and vomiting (RR = 2.00, 95%CI, 1.62–2.46, p<0.001), diarrhea (RR = 2.86, 95%CI, 1.95–4.19, p<0.001), nephrotoxicity (RR = 9.85, 95%CI, 2.27–42.70, p = 0.002), neurotoxicity (RR = 3.55, 95%CI, 1.41–8.97, p = 0.007). No statistically difference in thrombocytopenia (RR = 1.25, 95%CI, 0.85–1.85, p = 0.26) and toxic death (RR = 2.82, 95%CI, 0.75–10.59, p = 0.12). Nonetheless, the toxic death was almost three-fold higher in platinum-based therapy (Table 1).

**Table 1:** Comparison of Toxicity between Platinum Arms and Nonplatinum Arms with old-generation agents.

Toxicity	Number of Trials	Incidence of toxicity (%)		RR	95% CI	P
		Platinum Arm	Nonplatinum Arm			
anemia	8	24.25	12.55	1.93	[1.53, 2.44]	P<0.001
neutropenia	6	38.81	1.26	30.67	[16.92, 55.57]	P<0.001
thrombocytopenia	9	6.90	5.51	1.25	[0.85, 1.85]	P = 0.26
Febrile neutropenia	5	8.73	0.96	9.05	[3.64, 22.50]	P<0.001
Nausea and vomiting	10	21.62	10.81	2.00	[1.62, 2.46]	P<0.001
diarrhea	10	7.42	2.60	2.86	[1.95, 4.19]	P<0.001
neurotoxicity	9	2.15	0.60	3.55	[1.41, 8.97]	P = 0.007
nephrotoxicity	7	2.26	0.23	9.85	[2.27, 42.70]	P = 0.002
Toxic death	5	1.37	0.49	2.82	[0.75, 10.59]	P = 0.12

Abbreviation: RR, Risk Ratio; CI: Confidence Interval.

Platinum based therapy compared to non-platinum based therapy containing S-1 or taxanes or irinotecan was associated with a significantly increased risk for anemia (RR = 1.64; 95%CI, 1.31–2.04,p<0.001), neutropenia (RR = 2.40;95%CI, 2.03–2.83,p<0.001), thrombocytopenia (RR = 2.86;95%CI, 1.65–4.95,p<0.001) and febrile neutropenia (RR = 2.10 95%CI, 1.35–3.27,p<0.001).Similarly, the incidence of nausea and vomiting (RR = 2.12, 95%CI, 1.65–2.72,p<0.001)and neurotoxicity (RR = 2.01, 95%CI, 1.05–3.86, p = 0.03) appears to be higher in platinum arm. However, regarding the diarrhea, significant decrease was found in platinum arm compared to non-platinum arm with new generation agents (RR = 0.54, 95%CI, 0.38–0.75,p<0.001).Although the toxic death rate and the nephrotoxicity lacking statistical difference, it was noteworthiness that toxic death rate was about 5-fold higher (RR = 5.02, 95%CI, 0.59–42.69, p = 0.14)and nephrotoxicity was 4-fold higher (RR = 4.10, 95%CI, 0.89–18.90, p = 0.07) in the platinum arm (Table 2).

**Table 2:** Comparison of Toxicity Between Platinum Arms and Nonplatinum Arms with new-generation agents.

Toxicity	Number of Trials	Incidence of toxicity (%)		RR	95% CI	P
		Platinum Arm	Nonplatinum Arm			
anemia	9	23.4	14.3	1.64	[1.31, 2.04]	P<0.001
neutropenia	9	46.9	19.5	2.40	[2.03, 2.83]	P<0.001
thrombocytopenia	8	9.07	3.17	2.86	[1.65, 4.95]	P<0.001
Febrile neutropenia	7	8.56	4.07	2.10	[1.35, 3.27]	P<0.001
Nausea and vomiting	10	21.28	10.03	2.12	[1.65, 2.72]	P<0.001
diarrhea	8	6.88	12.83	0.54	[0.38, 0.75]	P = 0.002
neurotoxicity	9	37.7	18.7	2.01	[1.05, 3.86]	P = 0.03
nephrotoxicity	5	1.51	0.37	4.10	[0.89, 18.90]	P = 0.07
Toxic death	4	1.55	0.31	5.02	[0.59, 42.69]	P = 0.14

Abbreviation: RR, Risk Ratio; CI: Confidence Interval.

## Discussion

For decades, the chemotherapy containing platinum was almost generally used in Europe and United States, though there was no definitive evidence showing platinum should be backbone in palliative chemotherapy or a standard of care in patients with advanced gastric cancer. Numerous randomized phase II and phase III clinical trials have produced conflicting results. Additionally, the previous meta-analysis did not include the studies of comparing platinum with new or more active agents [3].Since the development of taxanes, irinotecan, S-1 and other new generational agents, the controversy has been more intense on whether the non-platinum-based can replace the platinum-based regimens without expense of reducing the response rate or overall survival. In Japan, S-1 was widely accepted both as monotherapy and combined therapy recently, but not in Europe or United states. Because of the limited sample sizes in single trial analysis, it was difficult to draw definitive conclusions.

This meta-analysis confirms that platinum-based regimens were more effective than the old-generation non-platinum based regimens, with an absolute improvement of 15% OS, but at the expense of higher toxicity in anemia, neutropenia, nausea and vomiting, diarrhea, nephrotoxicity and neurotoxicity. So evaluating patients’ state carefully before choosing platinum as chemotherapy regimens may benefit patients in the way of increasing response rate and overall survival.

Our study also provides the evidence that receiving new-generation agents instead of platinum does not cost reduction of efficiency in patients with advanced gastric cancer compared to platinum regimens. Furthermore, the quantitative evaluation of overall survival results suggests that the prognosis could not be improved in patients treated with platinum compared to non-platinum-based regimens containing new agents. Because of the opportunity of response not be impaired and the multiple toxicity of platinum, the decision of applying non-platinum regimens which contain new-generation drugs should be made especially when patients cannot tolerate the side effects of platinum. In addition, the new agents may be recommended to patients who were anxious to achieve life quality improvement. However, side effects of new agents and old generational drug differ significantly. Diarrhea was found obviously frequent, mainly because of irinotecan contained in non-platinum arm in some trials, which was consistent with previous knowledge. Although taxanes were involved in nerve impairment, no difference of neurotoxicity was observed which might be due to the fact that only grade 3 and 4 toxicity were collected. Besides that no other significant difference were established between the two non-platinum arms.

In our study, some limitations should be discussed. The analysis was not based on individual patient data because a lot of needed information could not be agreed to submit by authors, and this may overestimate treatment effects. Indeed, we conducted comparative randomized controlled trials as many as possible to provide robust estimates. The risk of publication bias exists but is not worth noticing in our analysis, because we included many positive and negative trials without language limitation. In our meta-analysis, we had dealt with numerous heterogeneity problems. Heterogeneity is a potential problem to affect results and was found among the included trials and subgroups. Although we choose to use the random effects model to calculate the estimates, the cause of heterogeneity should be explained. For example, the heterogeneity among trials could be caused by difference in patients’ characters, regimens and doses. Actually, we conducted inclusion criteria to make sure the similarity of patient selection and subgroup analysis to ensure the similar interventions, which reduced heterogeneity as much as possible.

### Conclusions

In conclusion, the platinum-based therapy may be challenged by new-generation agent based combination regimens because of similar response and overall survival. Furthermore, there seems to be more side effects in platinum-based regimens, including anemia, neutropenia, thrombocytopenia, febrile neutropenia, nausea and vomiting and neurotoxicity. Overall, the new generation agent based combination regimens can achieve the goal of tumor-shrinkage, similar effects on survival, and better tolerability. S-1, taxanes and irinotecan seem to be valid options for patients in first-line chemotherapy. Further evidence could be gained by meta-analysis of individual data.

## Supporting Information

Figure S1 **Assesing risk of bias for all eligible randomized controlled trials.**
(TIF)

Table S1 **Trails comparing platinum-based regimens with non-platinum-based regimens.**
(DOCX)
