# Supplementary figures and images for: Platinum-Based Versus Non-Platinum-Based Chemotherapy as First Line Treatment of Inoperable, Advanced Gastric Adenocarcinoma: A Meta-Analysis
Source: PLoS One. 2013 Jul 11;8(7):e68974. doi: 10.1371/journal.pone.0068974 (PMC3708886; doi:10.1371/journal.pone.0068974)

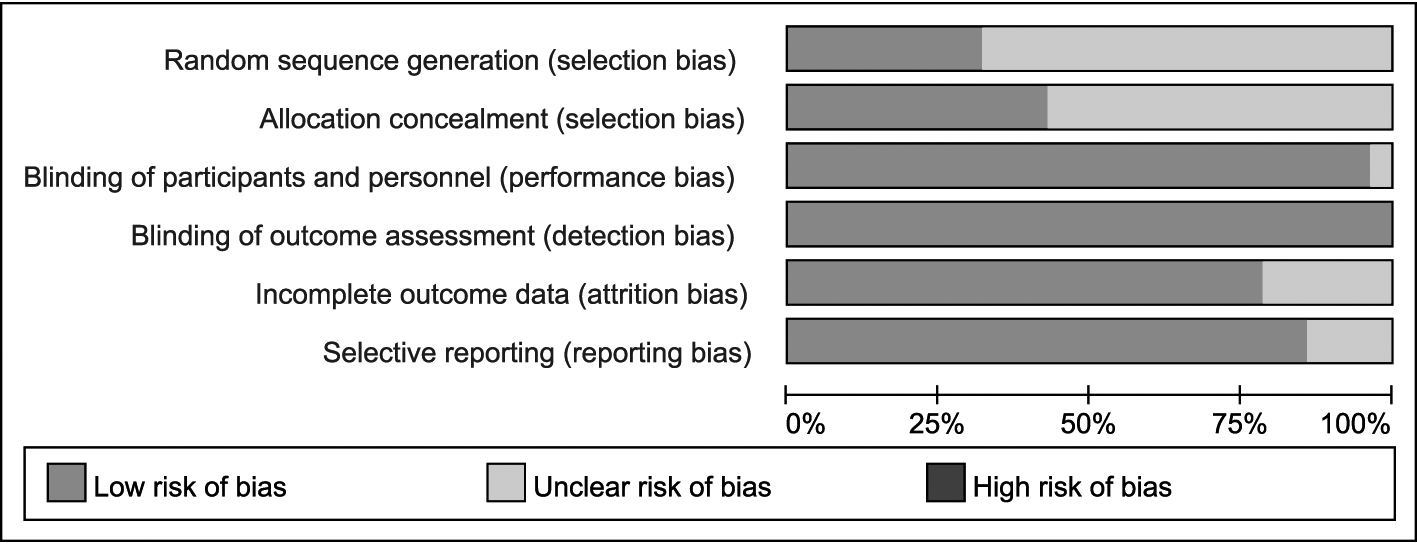

Supplement: Figure S1 — Assesing risk of bias for all eligible randomized controlled trials. (TIF) [file pone.0068974.s001.tif]
