# Supplementary material for: Platinum-Based Versus Non-Platinum-Based Chemotherapy as First Line Treatment of Inoperable, Advanced Gastric Adenocarcinoma: A Meta-Analysis
Source: PLoS One. 2013 Jul 11;8(7):e68974. doi: 10.1371/journal.pone.0068974 (PMC3708886; doi:10.1371/journal.pone.0068974)
Supplement: Table S1 — Trails comparing platinum-based regimens with non-platinum-based regimens. (DOCX) [file pone.0068974.s002.docx]

Table.S1 Trails comparing platinum-based regimens with non-platinum-based regimens

| Author and year | Publication form | Country  (Region) | Arm | No. of patients assessable for response (No. of response) | No. of patients assessable for survival | No. of patients assessable for toxicty | |
| --- | --- | --- | --- | --- | --- | --- | --- |
| Group GTS[^12^](#_ENREF_12),1988 | Full text | US | 5-fu-doxorubicin-triazinate  5-fu-doxorubicin-cisplatin  5-fu-doxorubicin-semustine | 31(6)  30(6)  33(5) | 81  85  81 | 81  85  81 | |
| Kikuchi [^14^](#_ENREF_14),1990 | Full text | Japan | 5-fu-adriamycin  5-fu-adriamycin-CDDP | 19(0)  18(6) | 33  32 | 33  32 | |
| Kelsen [^13^](#_ENREF_13),1992 | Full text | US | Etoposide-adriamycin-cisplatin  fluorouracil-adriamycin-leucovorin | 30(6)  30(10) | 30  30 | 30  30 | |
| Kim [^15^](#_ENREF_15),1993 | Full text | Korea | 5-fu-cisplatin  5-fu- doxorubicin-mitomycin  5-fu | 55(28)  57(14)  54(14) | 103  98  94 | 103  98  94 | |
| Cocconi [^8^](#_ENREF_8),1994 | Full text | Italy | Fluorouracil-doxorubicin-mitomycin  Cisplatin-epirubicin-leucovorin-fluorouracil | 52(8)  85(37) | 55  88 | N  N | |
| Cullinan [^10^](#_ENREF_10),1994 | Full text | US | 5-fu-doxorubicin-cisplatin  5-fu-doxorubicin-methyl lomustine  5-fu-doxorubicin-methyl lomustine-triazinate  5-fu | N  N  N  N | 51  53  79  69 | N  N  N  N | |
| De Lis [^11^](#_ENREF_11),1996 | Full text | Italy | 5-fu-doxorubicin-mitomycin  Cisplatin-doxorubicin-mitomycin | 50(13)  52(11) | N  N | 50  52 | |
| Barone [^5^](#_ENREF_5),1998 | Full text | Italy | 5-FU-6S-leucovorin  Epirubicin-etoposide-cisplatin-lonidamine | 33(6)  32(7) | 34  35 | N  N | |
| Vanhoefer [^19^](#_ENREF_19),2000 | Full text | Europe | Etoposide-leucovorin-fluorouracil  Flurorouracil-cisplatin  Flurorouracil-doxorubicin-methotrexate | 79(7)  81(16)  85(10) | 132  124  133 | 129  127  122 | |
| Cocconi [^9^](#_ENREF_9),2003 | Full text | Italy | Cisplatin-epirubicin-leucovorin-fluorouracil  Fluorouracil-doxorubicin-mitomycin | 98(38)  97(21) | 93  94 | 94  93 | |
| Bouche [^7^](#_ENREF_7), 2004 | Full text | France | leucovorin-flurorouracil  leucovorin-flurorouracil-Cisplatin  leucovorin-flurorouracil-Irinotecan | 45(6)  44(12)  45(18) | 45  44  45 | 45  44  45 | |
| Pozzo [^18^](#_ENREF_18),2004 | Full text | Europe | Irinotecan-5-FU)-folinic acid irinotecan-cisplatin | 74(25)  72(18) | 74  72 | 74  72 | |
| Tuss-Patience [^29^](#_ENREF_29),2005 | Full text | Germany | docetaxel-fluorouracil  epirubicin-cisplatin-fluorouracil | 45(17)  45(16) | 45  45 | 45  45 | |
| Yang [^30^](#_ENREF_30),2005 | Full text | China | Calcium folinate-5-FU-DDP  Calcium folinate-5-FU-Taxol  Taxol-oxalipatin | 53(22)  56(32)  57(37) | N  N  N | N  N  N | |
| Lutz [^16^](#_ENREF_16),2007 | Full text | Europe | HD-FU  HD-FU-FA  HD-FU-cisplatin | 33(2)  48(12)  46(21) | 37  54  54 | 37  53  51 | |
| Dank [^22^](#_ENREF_22),2008 | Full text | France | Irinotecan-5-fluorouraci  Cisplatin-5-fluorouraci | 170(54)  163(42) | 170  163 | 170  163 | |
| Popov [^17^](#_ENREF_17),2008 | Full text | Serbia | doxorubicin-etoposide-cisplatin  HD 5-FU | 30(10)  30(3) | 30  30 | N  N | |
| Tesselaar [^40^](#_ENREF_40),2008 | Abstract | Netherlands | 5-fluorouracil-leucovorin-cisplatin  5-fluorouracil-leucovorin-paclitaxel | 28(13)  27(12) | N  N | N  N | |
| Koizumi [^26^](#_ENREF_26),2008 | Full text | Japan | S-1  S-1 plus cisplatin | 106(33)  87(47) | 150  148 | 150  148 | |
| boku [^6^](#_ENREF_6) ,2009 | Full text | Japan | Fluorouracil  Irinotecan plus cisplatin  S-1 | 175(15)  181(68)  174(49) | 234  236  234 | 232  234  234 |  |
| Hou [^4^](#_ENREF_4),2009 | Ful text | China | Docetaxel-cisplatin-fluorouracil  Etoposide-calcium folina-fluorouracil | 19(10)  17(3) | 20  20 | 20  20 |  |
| Moehler [^28^](#_ENREF_28),2009 | Full text | Germany | capecitabine-irinotecan  capecitabine-cisplatin | 53(20)  50(21) | 53  50 | 57  55 |  |
| Jeung [^24^](#_ENREF_24),2010 | Full text | Korea | Docetaxel-cisplatin  Docetaxel-s-1 | 41(10)  39(18) | 41  39 | 41  39 |  |
| Kim [^25^](#_ENREF_25),2011 | Full text | Korea | Docetaxel-cisplatin  Irinotecan-leucovorin-5-fluorouracil | 28(7)  30(4) | 28  30 | 28  30 |  |
| Zhao [^31^](#_ENREF_31),2011 | Full text | China | Irinotecan-capecitabin  Oxaliplatin-capecitabin | 32(13)  31(12) | N  N | 32  31 |  |
| Maiello [^39^](#_ENREF_39),2011 | Abstract | Italy | Epirubicin-cisplatin-capecitabine  Docetaxel-5-FU | 36(20)  31(7) | N  N | 36  31 |  |
| Mochiki [^27^](#_ENREF_27),2012 | Full text | Japan | S-1-docetaxel  S-1-cisplatin | 42(22)  41(20) | N  N | 42  41 |  |

N：No available information
